# Effect of Muscle Energy Techniques V/S Active Range of Motion Exercises on Shoulder Function Post Modified Radical Neck Dissection in patients with Head and Neck Cancer - A Randomized Clinical Trial

**DOI:** 10.31557/APJCP.2020.21.8.2389

**Published:** 2020-08

**Authors:** Anmol Thomas, Cherishma D’Silva, Leah Mohandas, Sudeep M J Pais, Stephen Rajan Samuel

**Affiliations:** 1 *MPT Father Muller Medical College Hospital, Mangalore, Karnataka, India. *; 2 *Department of Physiotherapy, Father Muller Medical College Hospital, Mangalore, Karnataka, India. *; 3 *Department of Physiotherapy, Kasturba Medical College Mangalore, Karnataka, India. *

**Keywords:** Modified radical neck dissection, radical neck dissection, shoulder function, muscle energy techniques

## Abstract

**Purpose:**

Shoulder and cervical pain, reduced mobility and disability are some of the major complications associated with surgeries of head and neck cancers affecting several domains of quality of life. In the present study we aimed to compare the effectiveness of Muscle Ener-gy Techniques (METS) and Active Range of Motion Exercises in reducing pain, improving shoulder mobility and function in patients post Modified Radical Neck Dissection (MRND).

**Methods:**

Forty eight subjects were randomly assigned to two groups. Group A received active range of motion (AROM) exercises and group B received Muscle energy techniques (METS). Both the groups were treated for a period of 10 consecutive days starting from the 3rd to 5th postoperative day. Data was collected on the 1st and 10th day of intervention.

**Results:**

Both groups showed highly significant improvements in shoulder range of motion , decrease in pain and better Global Rating Change cores(GRCS) (p=0.005). GRCS and shoul-der abduction showed significant improvement in group B when compared to group A, sug-gesting better clinical outcomes in those treated with Muscle Energy Techniques.

**Conclusion:**

This study showed that both METs and AROM exercises were effective in im-proving shoulder range of motion, function and reducing pain in patients post MRND but-Muscle Energy Techniques were more effective when compared to AROM exercises.

## Introduction

Over 2 million cases of head and neck cancer occur each year in India. Head and neck cancer accounts for 30% of all cancers in India and nearly 50% deaths occur within 12 months of diagnosis. Nearly 50% of the survivors are left disabled due to surgery and chemotherapy (Guru et al., 2012).

Neck dissection followed by chemotherapy and radiotherapy is the treatment of choice for head and neck cancer (HNC) (Robbinsons et al.,1991) Radical neck dissection (RND) is one of the major treatment procedures used in the management of head and neck cancers but is known to have higher morbidity, greater post-operative complications and loss of function. It involves complete sacrifice of the sensory branches of the cervical plexus, and the C2, C3 and C4 branches that supply sternocleidomastoid and trapezius. This in turn leads to disfig-urement, which includes asymmetry of the mouth and facial oedema, loss of normal shoulder contour, abnormal scapular prominence and hollowing of the scapular region. Patients also complain of shoulder pain, sensory disturbances, and reduced strength of the arm and diffi-culty with their activities of daily living. To minimize these post-operative risks and compli-cations new and modified techniques such as selective neck dissection and modified radical neck dissection, have come into practice (MacComb,1968; Ewing and Martin,1952). Modified radical neck dissection (MRND), allows preservation of the spinal accessory nerve. In this procedure, some of the important structures are spared. MRND reduces the post-operative complications and disability to the neck and shoulder when compared to classic RND proce-dure. Even though spinal accessory nerve (SAN) is spared in MRND, there still persists high incidence of shoulder complaints and disability post-surgery. This is because preservation of SAN does not completely eliminate the risk of post-operative musculoskeletal complications. The incidence of shoulder complaints post RND surgery ranges from 47% to 100%, and it ranges from 18% to 77% post MRND (Wilgen et al., 2003;Wilgen et al., 2004; Ganne et al., 2017) EMG studies following MRND showed that the amplitude and the response area of evoked potential were significantly lower when compared to the non-operated side. Also the development of secondary complications such as joint fibrosis and secondary adhesive cap-sulitis can lead to reduced mobility at the shoulder. A recent study also showed that male patients with HNC are at risk of developing sarcopenia and its related adverse ef-fects (Chauhan et al., 2020) These impairments have a severe impact on the quality of life of these patients(Gang et al., 2017; Chepeha et al., 2002 ; Cheng et al., 2000; Luan et al., 2006).

Studies show that various physiotherapy modalities such as interferential therapy and faradic stimulation and techniques such as passive range of motion exercises, active assisted range of motion exercises, active range of motion exercises, strengthening exercises, PNF techniques, progressive resistance training (PRT), stretching exercises are known to prevent postoperative complications and also improve the shoulder range of motion there by reducing shoulder stiffness and tightness caused due to various causes (Baggi et al., 2014; Lauchlan et al., 2011; Ginn and Cohen, 2005; Reeve et al., 2010)

Muscle energy techniques (METS) are a set of soft tissue manipulation methods which include directed and controlled, isometric and/or isotonic contractions, which are performed by the patients. These techniques are aimed at reducing pain and improving musculoskeletal function (Bathia et al., 2016) Although both Active ROM exercises and MET exercises are known to improve shoulder function and pain post surgically, no studies were previously conducted to compare their effectiveness in this population. The aim of this study was to compare the effectiveness of these two treatment procedures and to determine which tech-nique would be effective in improving shoulder range of motion and reducing pain post MRND.

## Materials and Methods

*Methodology*

**Figure 1:**
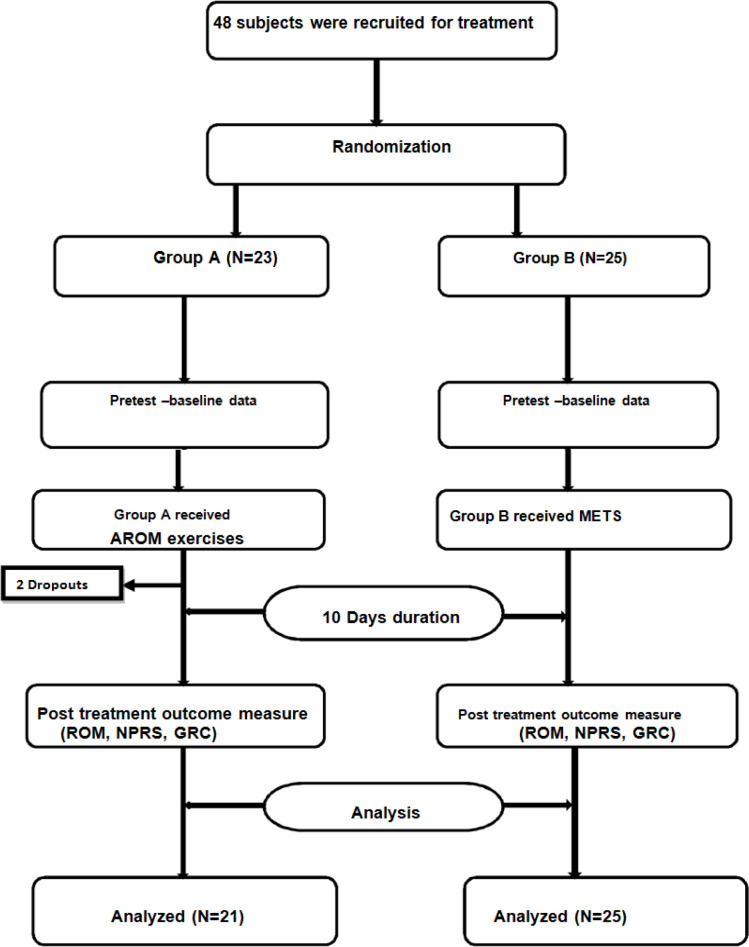
CONSORT Flow Chart

Ethical Clearance was obtained from the Institutional Ethical Committee. Forty eight subjects with a mean age of 53.64 years, who underwent MRND surgery were included. Patients will-ing to participate were explained about the study in their own language and written informed consent was obtained. Patients with Head and Neck Cancers who underwent recent MRND surgery ,aged between 30-65 years were included in the study. Patients were excluded if they had any 1) recent fracture or surgery to the shoulder 2) rotator cuff injury 3) recurrent shoul-der dislocation. Subjects were then randomly allocated into two groups using concealed enve-lope method (Figure 1).

The Baseline Measurements of ROM and Pain intensity were assessed and documented prior to the onset of treatment .The treatment began from 3^rd^ to 5^th^ postoperative day. Shoulder range of motion was measured using Universal goniometer and pain intensity was measured using numeric pain rating scale. Global rating of change scale (GRCS)which determines the effect of the intervention is a numerical rating scale , scoring between -5( very much worse) to + 5 (completely recovered), and 0 being unchanged. Group A received Active range of motion exercises (AROM) which included Pendulum exercises in standing position, active assisted ROM in supine position (which was then progressed to active exercises), cross body adduction in supine position and wall climbing or finger ladder exercises in standing posi-tion. Subject continued taking the arm up until the shoulder had maximum tolerable stretch, and was held at that position for 30 seconds. All exercises were given for 2 sets of 10 repetitions. Two subjects from Group A dropped out of the study as they developed post operative complications on the 4th post operative day. 

Group B received MET exercises which included post isometric relaxation techniques for shoulder flexion, abduction and internal rotation. Patient position was supine lying. They were asked to contract using 20% of their muscle force. The contraction was held for 7-10 seconds. Therapist then moved the shoulder passively into the range where a new restriction barrier was found. Pain, range of motion and GRCS were reassessed post 10 days of inter-vention.

## Results

A total of forty eight subjects were recruited for this study based on the inclusion and exclu-sion criteria. Two subjects from group A dropped out of the study as they underwent flap re-suturing and drain fixation during the intervention period. Following which results of forty-six subjects were analysed. Group A had a total of 21 subjects (15 males and 6 females). Group B had 25 subjects (22 males and 3 females). All subjects were similar at baseline with respect to age and the mean age of all subjects in group A was 52.90 (SD) and group B was 53.64 (SD) .NPRS at baseline showed a mean score of 7.10 ±0.83 and 7.24 ±0.59 in group A and group B respectively. Baseline scores of GRCS was -3.00 ±0.77 in Group A and and -3.60 ±0.50 in Group B. The baseline measures for shoulder abduction range of motion showed a mean value of 96.76±19.66 in group A and 92.16±10.61 in group B, internal rota-tion of 53.71±7.54 in group A and 54.16± 7.25 in group B, external rotation of 52.76± 8.97 in group A and 56.88± 6.16 in group B and flexion of 124.57±17.00 in group A and 123.04 ±14.64 in group B.

Results showed improvements in all shoulder ranges and pain in group A post AROM exer-cises. Shoulder abduction range increased from 96.76±19.66 to 112.00±19.98 (p=0.000), in-ternal rotation from 53.71±7.54 to 58.38±8.64 (p=0.000), external rotation from 52.76±8.97 to 57.90±9.56 (p=0.000) and flexion from 124.57±17.00 to 140.00±18.03 (p=0.000) which was highly significant. Also NPRS showed a reduction in pain from 7.10±0.83 to 4.33±1.19 (p=0.000) which was highly significant. GRCS also showed a significant improvement from -3.00 ±0.77 to 1.38±1.28.

Group B who received MET treatment also showed highly significant changes with respect to ROM pain and GRCS.The abduction range improved from 92.16±10.61 to 110.72 ±12.46 (p=0.000), internal rotation from 54.16±7.25 to 59.44±7.67 (p=0.000), external rotation from 56.88± 6.16 to 63.84±7.87 (p=0.000), flexion from 123.04±14.64 to 139.92±13.58 (p=0.000) ,NPRS from 7.24±0.59 to 4.20± 1.08(p=0.000) and GRC from -3.60 ±0.50 to 3.36±0.64.

Between group analysis was done to compare the improvements between group A and group B. It was found that group B who received MET treatment showed significant improvements when compared to group A in Abduction ROM (p=0.026), NPRS (p=0.282) and a highlyi sig-nificant improvement in GRC scores (p=0.000) when compared to Group A (Table 1 ).

**Table 1:** Pre Post Variables along with Mean, Standard Deviation, Difference of Mean & Standard Deviation, Percentage Change, Pre Post Significance and Significance between the Groups Group A, AROM; Group B, MET; NPRS, Numerical Pain Rating Scale; GRC, Global Rate Of Change Scales

Outcome Measure			Mean ±S.D.	Mean ±S.D of difference	Change (%)	Pre-Post significance	Significance between the groups
Abduction	Group A	Pre	96.76 ± 19.66	15.24±5.078	15.75	0.000 HS	0.026
		Post	112.00 ± 19.98				Sig
	Group B	Pre	92.16 ± 10.61	18.56±4.71	20.14	0.000 HS	
		Post	110.72 ± 12.46				
External-Rotation	Group A	Pre	52.76 ± 8.97	5.14±2.86	9.75	0.000 HS	0.08
		Post	57.90 ± 9.56				
	Group B	Pre	56.88 ± 6.16	6.96 ± 3.83	12.24	0.000 HS	NS
		Post	63.84 ±7.87				
Flexion	Group A	Pre	124.57 ± 17.00	15.43 ± 10.773	12.39	0.000 HS	0.533
		Post	140.00 ± 18.03				
	Group B	Pre	123.04 ± 14.64	16.88 ± 3.833	13.72	0.000 HS	NS
		Post	139.92 ± 13.58				
Internal Rotation	Group A	Pre	53.71 ± 7.54	4.67 ±2.033	8.69	0.000 HS	0.261
		Post	58.38 ± 8.64				
	Group B	Pre	54.16 ± 7.25	5.28 ± 1.621	9.75	0.000 HS	NS
		Post	59.44 ± 7.67				
NPRS	Group A	Pre	7.10 ± 0.83	2.76 ± 0.831	38.93	0.000 HS	0.282
		Post	4.33 ±1.19				
	Group B	Pre	7.24 ± 0.59	3.04 ±0.889	41.99	0.000 HS	NS
		Post	4.20 ± 1.08				
GRC	Group A	Pre	-3.00± 0.77	-4.38 ± 1.2	146.03	0.000 HS	0.000 HS
		Post	1.38 ± 1.28				
	Group B	Pre	-3.60 ± 0.50	-6.96± 0.89	193.33	0.000 HS	
		Post	-3.36 ± 0.64				

## Discussion

This study aimed at comparing the effectiveness of METs with AROM exercises in subjects who underwent MRND. All subjects recruited for this study showed a significant improve-ment in shoulder range of motion and also experienced a reduction in pain and felt clinically better after a treatment of 10 days. 

Significant improvements were found in shoulder range of motion and reduced pain post 10 days intervention period in subjects allocated to AROM (group A) and MET groups(group B). AROM exercises cause joint distraction as well as oscillations which results in decreased pain, early mobilization of joint and increased flow of nutrients to the joint space (Baggi et al., 2014 ; Ellsworth et al., 2006). Group B demonstrated significant improvements in shoul-der ranges and decreased intensity of pain compared to Group A. This could be due to the analgesic effect of MET allowing the subjects to reach greater range of motion while provid-ing greater stretch tolerance (Phadke et al., 2016; Moore et al., 2011; Gary and Christian , 2010). Our findings are similar to the study done by Narayan et al., (2014) which stated that MET exercises can improve the mobility of joints as it causes restoration of normal length- tension relationship of the muscles which in turn undergo adaptive or protective shortening due to intrinsic or extrinsic factors. Improvement in range can also be due to the reflex relaxation of the agonist group of muscles after an isometric contraction. This reflex relaxation is suggested to be mediated by the Golgi tendon organ and its inhibitory effect on the alpha-motor neuron pool. Reciprocal inhibition from the antagonist muscle contractions could also have an effect on improving the mobility of the joint (Chaitow, 2006 ; Magnusson et al., 1996) The changes in the muscle extensibility and joint range of motion could be related to the mechanisms which promote hypoalgesia and an increase in stretch tolerance of the subjects. However, in the current study we found that range of motion in abduction significantly had better gains in group B than in group A. MET have shown to improve muscle extensibility and also cause an increase in muscle strength with isometric contractions. The subjects in group B may have gained strength in abductors and this could be the reason for increase in abduction range of motion in group B. Also, there was an improvement in shoulder function in Group B which can be attributed to one of the effects of METs (Chaitow, 2006; Wilson et al., 2003; Ballantyne et al., 2003). 

Subjects of both the groups were on analgesics, on an average 4-5 days postoperatively, but the decrease in pain cannot solely be attributed to effects of the analgesics. Intensity of Pain (NPRS) decreased post intervention significantly in Group A and B. The exact physiology be-hind the therapeutic effects of MET on pain reduction is still not clear but it is suggested that it might be due to the involvement of various biomechanical and neurological mechanisms, such as altered proprioception, hypoalgesia, changes in tissue fluid, motor programming and motor control (Gary and Christian, 2010).

Between group analysis evaluated using Global rating change scores demonstrated an overall clinical progress in both the groups when compared to their baseline values .Group B demonstrated significantly better improvements than group A, this could be attributed to the fact that GRC scores are well correlated with pain, disability and Quality of life. Reduction in pain and improvement in range of motion in MET group could have lead to better outcomes (Kamper et al., 2009).

Significant improvements were also found in shoulder range of motion post 10 days inter-vention period in subjects allocated to AROM group (group A). AROM exercises cause joint distraction as well as oscillations which results in decreased pain, early mobilization of joint and increased flow of nutrients to the joint space (Baggi et al., 2014; Ellsworth et al., 2006).

In conclusion, the present study showed that Muscle energy technique (MET) and active range of motion (AROM) exercises are effective in improving shoulder range of motion , reducing pain and improved GRCS. Between group analyses demonstrates MET exercises to have better clinical outcomes in improving abduction range of motion, decreasing pain and clinically better global rating scorescompared to AROM exercises. 

Further studies with longterm followup and larger sample size could give better understand-ing.
